# Measuring the quality of maternal and care processes at the time of delivery in sub-Saharan Africa: development and validation of a short index

**DOI:** 10.1186/s12884-019-2281-z

**Published:** 2019-04-16

**Authors:** Vandana Tripathi, Cynthia Stanton, Donna Strobino, Linda Bartlett

**Affiliations:** 1 Department of Population, Family Planning, and Reproductive Health, Johns Hopkins Bloomberg School of Public Health, 615 N Wolfe St, Baltimore, MD 21205 USA; 2 EngenderHealth, 505 9th St NW, Washington, DC 20004 USA; 3 Department of International Health, Johns Hopkins Bloomberg School of Public Health, 615 N Wolfe St, Baltimore, MD 21205 USA

**Keywords:** Quality of care, Labor and delivery care, Postpartum care, Newborn care, Sub-Saharan Africa, Measure development

## Abstract

**Background:**

There is a growing recognition that quality of care must improve in facility-based deliveries to achieve further global reductions in maternal and newborn mortality and morbidity. Better measurement of care quality is needed, but the unpredictable length of labor and delivery hinders the feasibility of observation, the gold standard in quality assessment. This study evaluated whether a measure restricted to actions at or immediately following delivery could provide a valid assessment of the quality of the process of intrapartum and immediate postpartum care (QoPIIPC), including essential newborn care.

**Methods:**

The study used a comprehensive QoPIIPC index developed through a modified Delphi process and validated by delivery observation data as a starting point. A subset of items from this index assessed at or immediately following delivery was identified to create a “delivery-only” index. This delivery-only index was evaluated across content and criterion validation domains using delivery observation data from Kenya, Madagascar, and Tanzania, including Zanzibar.

**Results:**

The delivery-only index included 13 items and performed well on most validation criteria, including correct classification of poorly and well-performed deliveries. Relative to the comprehensive QoPIIPC index, the delivery-only index had reduced content validity, representing fewer dimensions of QoPIIPC. The delivery-only index was also less strongly associated with overall quality performance in observed deliveries than the comprehensive QoPIIPC index.

**Conclusions:**

Where supervision resources are limited, a measure of the quality of labor and delivery care targeting the time of delivery may mitigate challenges in observation-based assessment. The delivery-only index may enable increased use of observation-based quality assessment within maternal and newborn care programs in low-resource settings.

## Background

Maternal deaths have decreased dramatically in the past two decades; however, only nine of the 75 countries monitored by the Countdown to 2015 group have achieved the Millennium Development Goal (MDG) for reducing maternal mortality [1–4]. The lifetime risk of maternal mortality in sub-Saharan Africa is 1 in 39, but only 1 in 3800 in high income countries [3]. Two million intrapartum stillbirths and intrapartum event-related early neonatal deaths also continue to occur annually [5].

More women are delivering in facilities in many low-income countries. Contact with health care providers does not, however, guarantee that appropriate interventions will be provided during labor & delivery (L&D) and the immediate postpartum period, including essential newborn care (ENC) [6–8]. High quality care ensures that women and neonates receive interventions shown to reduce intrapartum and postpartum complications or to be effective in managing these complications [9–12]. Studies indicate that coverage with effective interventions is poor during the intrapartum and immediate postpartum periods [13–16]. Studies from multiple countries indicate that increasing facility delivery may not suffice for mortality reduction in settings with low quality of care (QoC) [17–19].

Assessing the quality of maternity services is challenging. The vast majority of deliveries are uncomplicated, yet obstetric complications may arise even when evidence-based care has been provided [8, 20, 21]. It is, therefore, essential to assess QoC not just through clinical outcomes, but also through evaluation of care processes during labor, delivery, and the immediate postpartum period [22, 23].

A recent literature review of indicators used to assess the quality of L&D care found hundreds of proposed indicators but little validation or standardization of measures [24]. There is no consensus about measurement of the quality of the process of intrapartum and immediate postpartum care (QoPIIPC), i.e., the actions conducted by providers during L&D care. Most existing tools to assess care processes have only been evaluated using expert opinion. Measures of QoPIIPC based on clinical guidelines or programmatic evidence can be lengthy; some include hundreds of indicators [25, 26]. Administering these tools is difficult and has significant opportunities for measurement error. A number of studies have assessed QoPIIPC through criterion-based audit or other record review, but generally relied on routine data sources, such as maternity registers, that are not designed for quality assessment. The indicator review also found that two-thirds of quality assessment studies, including nearly all criterion-based audits, focused on adverse events and maternal complications [24]. For example, the widely-used UN process indicators for maternal health programs target emergency obstetric and neonatal care (EmONC) [27, 28]. There is relatively little information about the quality of routine L&D care and ENC.

The indicator literature review also found that few studies have used observation of maternity care in assessing quality [24]. A substantial body of research suggests the unique role of direct observation in quality assessment [29, 30]. Numerous studies in low-resource settings have shown that facility records may not document actions that were performed and are otherwise incomplete and unreliable [29–32]. Several studies have shown low agreement among peers after reviewing the same records, particularly for indicators of care processes [33–35], and limitations to quality assessment using other non-observation methods such as vignette or case simulation [30].

The infrequent use of clinical observation in maternity services is understandable; the length of an episode of L&D care is unpredictable, with even uncomplicated cases having the potential to last up to 24 h [36]. Procuring skilled, expert observers can also be challenging in settings where the availability of providers is limited and workloads are high. The burden in obtaining observation data is a significant barrier to comprehensive assessment of L&D care in settings without adequate human, transport, and financial resources for supervision activities [37, 38]. A recent study developed and validated a comprehensive measure assessing actions throughout an episode of L&D care [39] This measure is the first empirically validated observation-based tool to assess QoPIIPC that we ae aware of. However, it faces limitations in use due to these burdens in observing an entire episode of L&D care.

These challenges notwithstanding, improved assessment of the quality of routine L&D services at health facilities is essential in the current era of rapidly increasing facility delivery. Robust quality measures must be valid and reliable, but also efficient. Observation-based tools in particular must minimize the burden on clinical supervisors in low-resource settings. To examine whether this burden could be reduced while maintaining the validity of quality measurement, this study evaluated whether a measure restricted to actions performed at and immediately after delivery can provide a meaningful assessment of QoPIIPC in facility-based L&D care in sub-Saharan Africa [39]. The current study sought to validate a measure focused on the time of delivery using the same data and validation criteria as the earlier, comprehensive index developed by the same study team.

## Methods

### Selection of index items

The current study used the comprehensive facility-based QoPIIPC index developed through earlier analysis as a reference point. The process of developing and validating the comprehensive index is briefly summarized here and has been reported in detail previously [39]. The comprehensive measure was developed following a modified Delphi process with maternal and neonatal care (MNC) experts to identify consensus dimensions of QoPIIPC. MNC experts also rated the ability of items, i.e., actions during intrapartum and postpartum care, to reflect these dimensions. The five consensus QoPIIPC dimensions identified by the expert group were technical quality, screening and monitoring quality, interpersonal care quality, the quality of infection prevention/control, and the avoidance of harmful or non-indicated interventions [39]. Indices containing combinations of highly-rated items were developed based on MNC expert ratings and evaluated for face, content, and criterion validity. Secondary data obtained from surveys observing L&D care at health facilities in sub-Saharan Africa were used in index validation. The comprehensive QoPIIPC index of 20 items was selected based on comparison of performance on several validation benchmarks [39]. The secondary data source and validation benchmarks are described further below.

For the analysis reported in this paper, the 20 items in the comprehensive QoPIIPC index were evaluated for whether they could be assessed at or immediately following delivery, thus avoiding observation of client intake and the unpredictably long first stage of active labor and early second stage of labor. Items meeting these criteria were retained in a “delivery-only” index.

### Secondary data source

The Maternal and Child Health Integrated Program (MCHIP), a USAID-funded global project implemented by Jhpiego, conducted the QoC Assessments, a set of observational surveys in sub-Saharan Africa from 2010 to 2013. QoC Assessment data were used to evaluate the delivery-only index were obtained from a series of observational surveys of QoC in sub-Saharan Africa between 2010 and 2013. Specifically, the study used data from QoC Assessments conducted in 2010–2011 in Kenya, Madagascar, and Tanzania, including Zanzibar; as well as a repeat survey in Tanzania alone in 2012–2013. The countries were selected due to similarity in their maternal health services and indicators [40–42].

As described in reporting the earlier study to develop a comprehensive QoPIIPC index [39], a structured checklist was used for delivery observations in the QoC Assessments, based on World Health Organization recommendations and other global guidelines and surveys [7, 15, 20, 43, 44]. The checklist included items about essential L&D care as well as care for maternal and newborn complications [45]. There were 131 routine care L&D items in the L&D observation checklist [39, 45].

The QoC Assessment sample sizes, at least 250 deliveries in each country, were intended to provide national estimates of routine L&D care practices. Details of sampling approaches and data collection tools are provided in each country’s survey report [45]. Analytic samples in this study were restricted to L&D cases observed across intake, active labor, delivery, and the immediate postpartum period. The Zanzibar and Round 1 Tanzania samples were merged for analysis, as the number of deliveries observed in Zanzibar was small. Data were not weighted for analysis.

### Observed delivery scores

The delivery-only index was evaluated within each country and across countries; it was compared to the comprehensive QoPIIPC index using QoC Assessment delivery observation data. As in the prior study, each observed delivery was assigned a comprehensive index score and a delivery-only index score. Each index item had a value of 1 if performed and 0 if not performed. These item scores were summed to create comprehensive and delivery-only index scores for each delivery. A total QoC score was also given to each delivery based whether each routine intrapartum and immediate postpartum care item in the full L&D observation checklist was performed.

### Validation domains and benchmarks

The delivery-only index was assessed across six validation domains, each with multiple benchmarks. The domains were: representation of QoPIIPC dimensions; association of the index score with overall QoC performance; relation of each item in the index to overall QoC performance; ability to discriminate between poorly and well-performed deliveries; inclusion of items that ranged in frequency of performance; and variability and distribution of the index score. These validation domains evaluate the degree to which an index measures and is informative about QoPIIPC. Benchmarks are specific, quantifiable, and comparable criteria within each validation domain. A total of 28 benchmarks were assessed across the six validation domains. Validation domains, benchmarks, and selection criteria are identical to those used to validate the comprehensive QoPIIPC index in the earlier study, and have been described previously [39]. A threshold of *p* < 0.05 was used in tests of statistical significance.

A particular focus of assessment was content and criterion validity. Content validity describes how well the index represents QoPIIPC, specifically the consensus dimensions identified through the Delphi process described above. Criterion validity is reflected by the relation of the index score to a reference measure of QoPIIPC. In this analysis, the total QoC score across all routine care items served as the reference measure of overall QoC performance.

To be useful, a quality measure must be able to discriminate between poorly and well-performed deliveries. Therefore, this domain accounted for a substantial proportion (15 of 28) of the validation benchmarks. To enable assessment of QoC discrimination, level of care quality was described with three dichotomous variables. First, relatively good performance was defined as being in the top 25% of the total QoC score distribution. Second, absolute good performance was defined as achieving at least 80% of the maximum possible total QoC score. Finally, relatively poor performance was defined as being in the bottom 25% of the total QoC score distribution. The three dichotomous variables were treated as the dependent variables in separate analyses.

Simple logistical regressions assessed the relation between index scores and the odds of being in each good/poor performance group. The area under receiver operating characteristic (AUROC) curves based on the logistic regression results was calculated for each good/poor performance classification. AUROCs indicate the ability of the index to correctly classify QoC. If two deliveries are drawn from the sample at random, the AUROC represents the proportion of pairs in which the delivery with the higher index score is in the good performance group and vice versa, for classification of poor performance. An AUROC of 0.7–0.9 shows moderate discrimination while over 0.9 is considered excellent discrimination [46, 47]. Predicted probabilities were calculated based on logistic regressions, representing the likelihood of being in the relative and absolute good performance groups at each value of the index score.

### Index comparison

Analyses also compared the performance of the delivery-only and comprehensive QoPIIPC indices. AUROC comparisons assessed the relative ability of each index to classify deliveries as good or poor performance. Likelihood ratio tests compared the fit of linear and logistic regression models of the association between index scores and overall QoC performance. Likelihood ratio test assessment was possible because the delivery-only index items were a subset of the comprehensive index items. Comparisons used standardized index scores to avoid differences due to the number of items included in the two indices.

To enable comparison between the delivery-only and comprehensive indices, performance on each validation benchmark was given a score for each index; the index that performed better on each benchmark received 1 point and the other, 0 points. The scores were summed for each domain. The index with a higher score within each validation domain received 1 point and the other, 0 points. Finally, validation performance scores summing across domains (potential range from 0 to 6, with 1 point for each domain) were calculated for each index within each country and across countries.

Because the comprehensive QoPIIPC index was developed through an extensive expert review and validation process, the aim of this analysis was not to determine whether the delivery-only index is a “better” measure of quality. Instead, comparative evaluation of validation performance sought to examine whether the delivery-only index may be a robust alternative in settings of limited resources for quality assessment and observation of care.

### Ethics and consent

The QoC Assessment protocol was reviewed and approved by ethical review boards in each country where the survey was conducted. In the countries whose data are analyzed in this study, these boards were: the Kenya Medical Research Institute Institutional Review Board (IRB) in Kenya; the Ministry of Health Ethical Committee in Madagascar; and the National Institute of Medical Research Institutional Review Board IRB in Tanzania. The Johns Hopkins Bloomberg School of Public Health IRB ruled the protocol for the QoC Assessment study across all countries exempt from review (reference number 00002549).

Written informed consent was obtained from facility directors prior to the QoC Assessment implementation. During data collection, verbal informed consent was obtained from providers and patients or patients’ next of kin. Providers were not asked to give written consent during the provision of L&D care; however, a comprehensive discussion of benefits and burdens was held with the facility directors in a non-service provision context. Patients or next of kin were not asked to provide written consent both because of literacy limitations and to reduce the burden on women during L&D. Verbal consent was recorded in the QoC Assessment data entry applications; each module of questions noted that provider and patient (or next of kin) consent was required before items in that module could be completed. Consent procedures were described in research plans submitted to and approved by the aforementioned IRBs. The names of individual patients and providers were not collected during service observations. The quantitative analyses reported in this study were conducted using secondary data without identifiers.

## Results

Deliveries observed across admission, active labor, and immediately postpartum were retained in analysis. This resulted in the inclusion of approximately two-thirds of observed deliveries from Kenya and Madagascar (626 and 347, respectively) but only 39–40% of deliveries in Tanzania/Zanzibar (706 in Round 1, and 558 in Round 2). However, there were almost no significant differences between the full sample and the analytic sample, in terms of women’s characteristics or provider and facility type [39]. Ultimately, approximately half the deliveries observed across the QoC Assessments were included in analysis – 1115 of 2237 deliveries across 310 health facilities. This is identical to the sample used in our earlier study to develop a comprehensive QoPIIPC index [39].

Table 1 lists the items in the comprehensive QoPIIPC index and delivery-only indices. The proportions of deliveries in which these items were performed in each country are described elsewhere [39].

**Table 1:** Items in the comprehensive and delivery-only indices^a^

Indicator	Comprehensive Index	Delivery-only Index
Checks woman’s HIV status (checks chart or asks woman) and/or offers woman HIV test	+	–
Asks whether woman has experienced headaches or blurred vision	+	–
Asks whether woman has experienced vaginal bleeding	+	–
Takes blood pressure	+	–
Takes pulse	+	–
Washes his/her hand before any examination	+	–
Wears high-level disinfected or sterile gloves for vaginal examination	+	–
At least once, explains what will happen in labor to the woman and/or her support person	+	+
Uses partograph (during labor)	+	+
Prepares uterotonic drug to use for AMTSL	+	+
Self-inflating ventilation bag (500 mL) and face masks (size 0 and size 1) are laid out and ready for use for neonatal resuscitation	+	+
Correctly administers uterotonic (timing, dose, route)	+	+
Immediately dries baby with towel	+	+
Places newborn on mother’s abdomen skin-to-skin	+	+
Ties or clamps cord when pulsations stop, or by 2–3 min after birth (not immediately after birth)	+	+
Assesses completeness of placenta and membranes	+	+
Assesses for perineal and vaginal lacerations	+	+
Takes mother’s vital signs 15 min after birth	+	+
Palpates uterus 15 min after birth	+	+
Assists mother to initiate breastfeeding within one hour	+	+

^a^If an item is in an index this is signified by ‘+.’ If an item is not in an index, this is signified by ‘-’

Table 2 provides illustrative results on validation benchmarks for both indices, based on the Tanzania Round 1 delivery observation data. The 13 items in the delivery-only index represented 3 of the 5 consensus QoPIIPC dimensions: technical quality, screening and monitoring, and interpersonal care. This is fewer than the 4 dimensions represented in the comprehensive index because both items for infection prevention were eliminated by restriction to the time of delivery. Five of the items for screening, monitoring, and the readiness to take action in case of danger signs were also eliminated in the delivery-only index.

**Table 2:** Comparison of comprehensive and delivery-only indices using Tanzania (including Zanzibar) Round 1 data^a^

	Comprehensive index	Delivery-only index
Score distribution		
Mean (% of maximum achievable)	12.12 (57.71%)	7.48 (57.54%)
Maximum (% of maximum achievable)	21 (100.00%)	13 (100.00%)
Minimum (% of maximum achievable)	0 (0.00%)	0 (0.00%)
Validation domains and benchmarks		
1. Representation of QoPIIPC dimensions:		
- # of dimensions (out of 5)	4	3
2. Association of index with overall QoC performance:		
- B coefficient from SLR of total QoC score (p-value)	8.91^b^ (< 0.001)	8.20 (< 0.001)
3. Association of individual items with overall QoC performance:		
- # items without significant relationship to total QoC score	1	0
- # items without significant relationship to good QoC score (absolute)	4	3
- # items without significant relationship to poor QoC score (relative)	3	1
4. Ability to distinguish between good and poor performance:		
- AUROC good total QoC score - absolute	0.976^c^	0.924
- AUROC good total QoC score – relative	0.935	0.918
- AUROC poor total QoC score - relative	0.940^c^	0.900
- OR good total QoC score – absolute (*p*-value)	51.33 (*p* < 0.001)	10.00 (*p* < 0.001)
- OR good total QoC score – relative (p-value)	34.08^d^ (*p* < 0.001)	16.43 (*p* < 0.001)
- OR poor total QoC score – relative (*p*-value)	0.029^d^ (*p* < 0.001)	0.072 (*p* < 0.001)
5. Range of performance frequency:		
- # of items performed in < 30% of cases	3	2
- # of items performed in < 40% of cases	5	3
- # of items performed in > 90% of cases	3	2
6. Distribution of index score:		
- Coefficient of variation	28.52	30.73
- % of deliveries with minimum index score	0.35%	0.71%
- % of deliveries with maximum index score	0.71%	1.77%

^a^Standardized coefficients and ORs are presented to enable comparison across indices with different numbers of items

^b^Significant difference from coefficient for delivery-only index (likelihood ratio test)

^c^Significant difference from AUC for delivery-only index (χ^2^)

^d^Significant difference from OR for delivery-only index (likelihood ratio test)

The delivery-only index score showed a statistically significant association with the total QoC score across all country samples, with an increase of 2.80 to 3.09 points in the total score with each one-point increase in the index score. This association indicates that performing one additional intervention included as an item in the delivery-only index was associated with performance of several additional best-practice interventions during the full episode of L&D care.

**Fig. 1:**
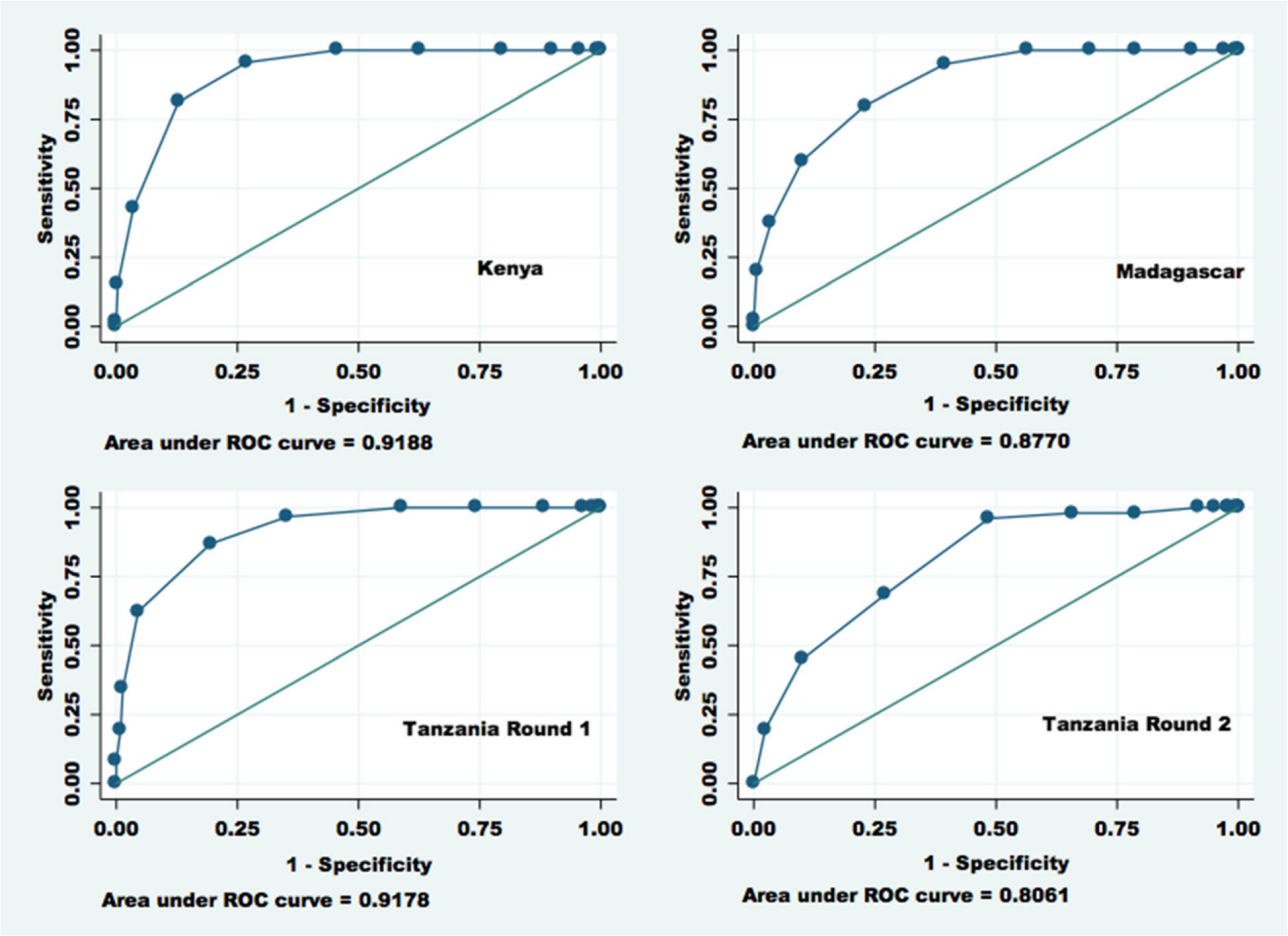
AUROCs (discrimination of good total quality score (top 25%)): Delivery-only index

An increasing delivery-only index score was associated with significantly increased odds of being in the good performance category for total QoC, whether defined absolutely or relatively. This finding was consistent across countries. Similarly, an increasing index score was associated with significantly decreased odds of being in the poor performance category for total QoC (see Table 2 for illustrative results), across countries. The delivery-only index showed moderate to excellent ability across countries to distinguish between good and poor performance. AUROCs ranged from 0.913 to 0.927 in Kenya, from 0.877 to 0.931 in Madagascar, from 0.900 to 0.924 in Tanzania Round 1, and from 0.806 to 0.833 in Tanzania Round 2. AUROCs were generally lower for classifying cases into the poor performance category. Figure 1 describes AUROCs for identification of delivery cases in the relative good performance group (top 25% of the total QoC score distribution). The results indicate that, for instance, if two deliveries were randomly drawn from the Tanzania Round 1 sample, in 92% of these pairs, the delivery-only index would correctly classify care quality, i.e., the case with the index score would be in the good performance group.

**Fig. 2:**
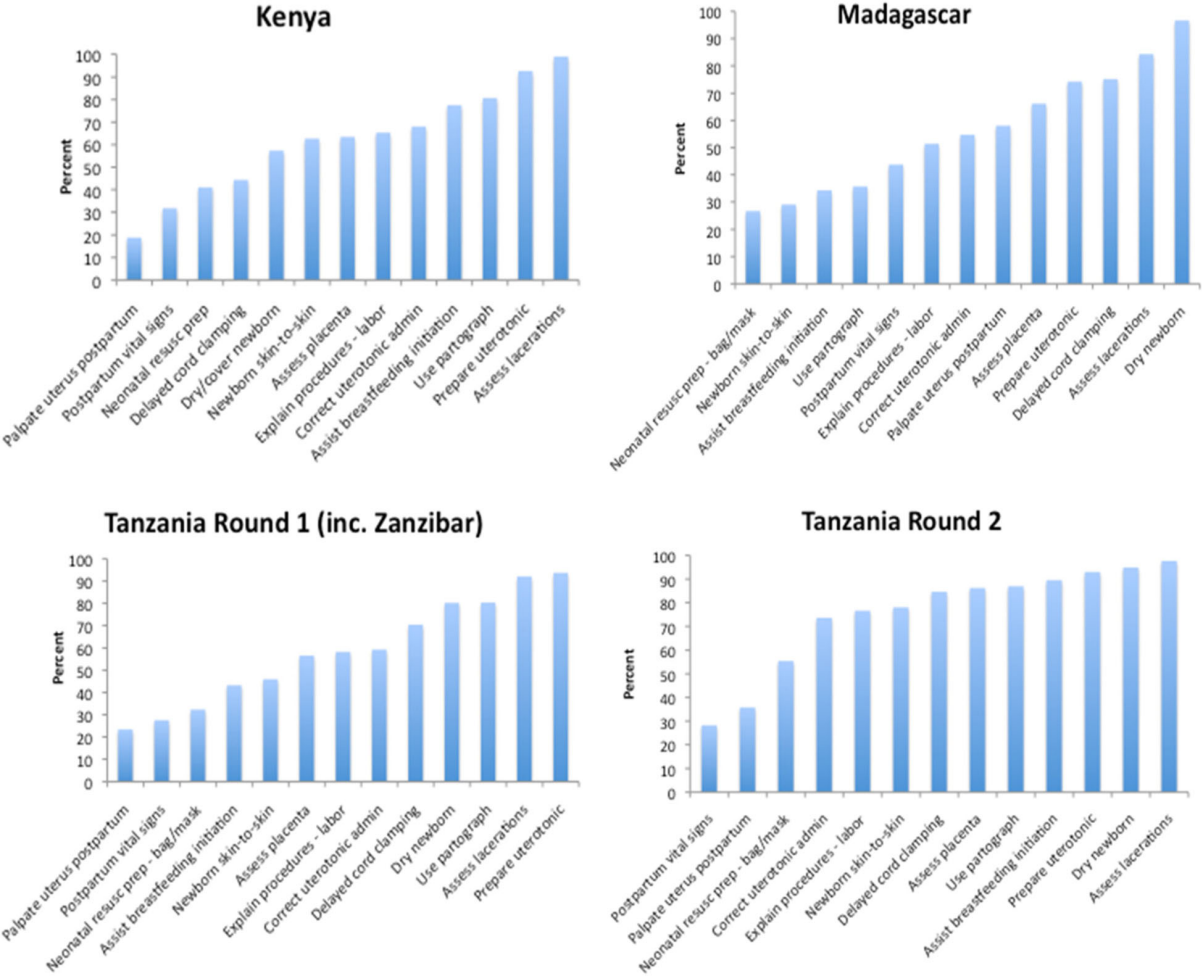
Performance of delivery-only index indicators across countries

Figure 2 shows the frequency with which delivery-only index items were performed across countries. Ceiling or floor effects were not observed in the distribution of index scores. Across countries, 1–2 items were performed correctly in under 30% of cases, and 1–3 items were performed correctly in over 90% of cases.

**Fig. 3:**
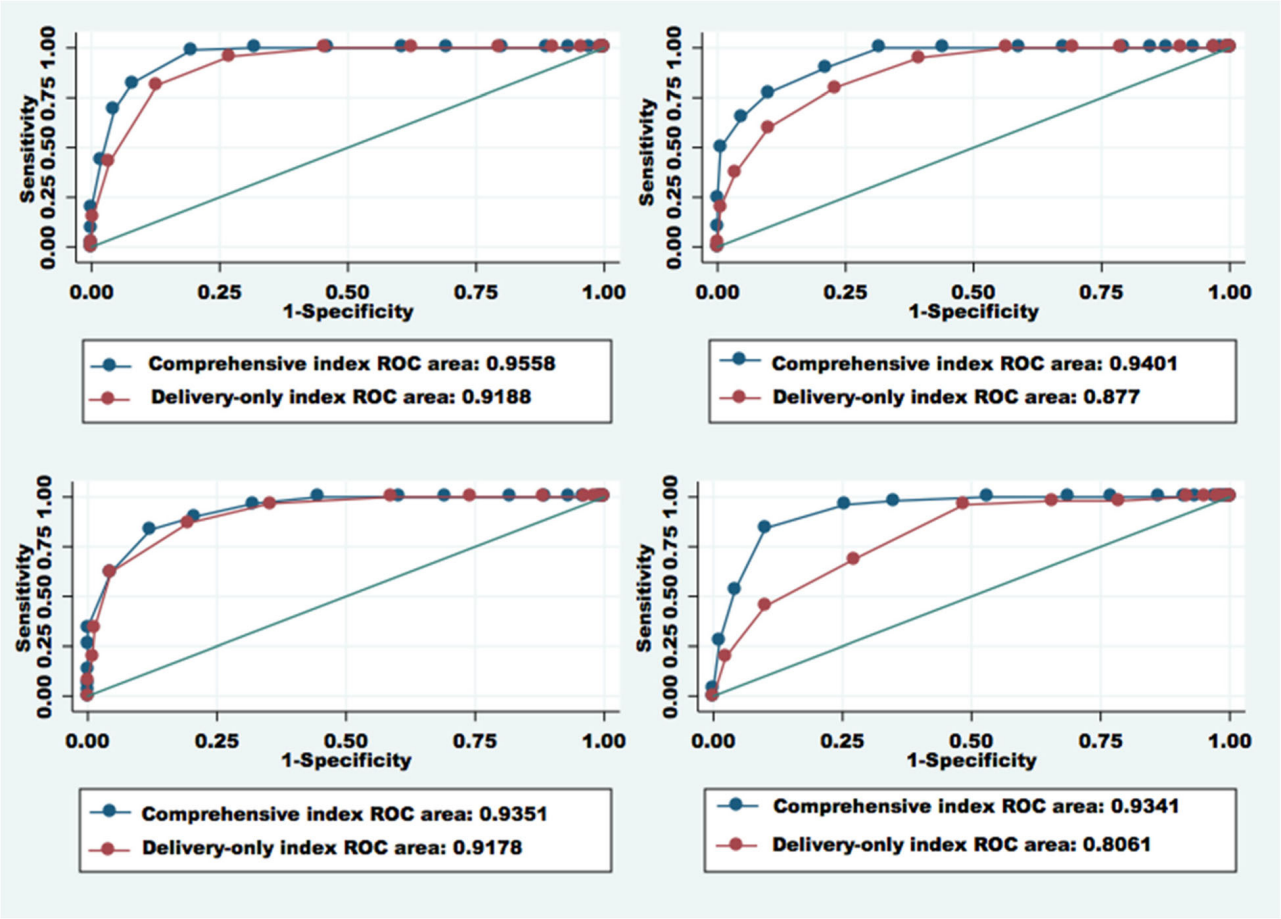
AUROCs (discrimination of good total quality score (top 25%)): Comparison of the comprehensive and delivery-only indices

The delivery-only index performed well on most measures of content and criterion validity. However, comparison with the comprehensive QoPIIPC index (see Tables 2 and 3) showed that the magnitude of the association with the total QoC score and the ability to distinguish poorly and well-performed deliveries were attenuated for the delivery-only index. Figure 3 compares the AUROCs for both indices, indicating the stronger ability of the comprehensive QoPIIPC index to classify deliveries as well or poorly performed. While statistically significant across most comparisons, this difference was larger in Madagascar and Tanzania Round 2. Based on all likelihood ratio tests comparing linear and logistic regression models of the relation between the index score and total QoC score, the comprehensive QoPIIPC index also fit the data better than the delivery-only index.

**Table 3:** Summary of index performance across validation domains^a^

	Comprehensive index	Delivery-only index
Kenya		
Dimension representation	1	0
Association with overall QoC	1	0
Discrimination of good/poor performance	1	0
Item association with overall QoC	1	1
Item performance range	1	1
Variability and distribution of index score	1	0
Total	6	2
Madagascar		
Dimension representation	1	0
Association with overall QoC	1	0
Discrimination of good/poor performance	1	0
Item association with overall QoC	0	1
Item performance range	1	0
Variability and distribution of index score	1	1
Total	5	2
Tanzania R1 (incl. Zanzibar)		
Dimension representation	1	0
Association with overall QoC	1	0
Discrimination of good/poor performance	1	0
Item association with overall QoC	0	1
Item performance range	1	0
Variability and distribution of index score	0	1
Total	4	2
Tanzania R2		
Dimension representation	1	0
Association with overall QoC	1	0
Discrimination of good/poor performance	1	0
Item association with overall QoC	0	1
Item performance range	0	1
Variability and distribution of index score	0	1
Total	3	3
Total across countries	18	9

^a^Each index received 1 point if it was the better performing on the measures of a particular domain; ties were acceptable

Notably, the delivery-only index performed better than the comprehensive QoPIIPC index on several validation benchmarks in some or all countries, including: having fewer items with no statistically significant association with the total QoC score, a better range of frequency with which index items were performed (fewer “easy” items and more “difficult” items), and a greater coefficient of variation.

The predicted probabilities of being in the relative (top 25% of the total QoC score distribution) and absolute (≥80% of possible items performed correctly) good performance group at each value of the delivery-only index score are provided in Table 4, using Tanzania Round 1 data. For example, the probability of being in the relative good performance group is just 4% at the mean delivery-only index score (7). There is a substantial increase in the likelihood of good performance with each one-point increase in the index score above this mean. These patterns are comparable to those in the predicted probabilities of good performance at each level of the comprehensive QoPIIPC index score, as reported previously [39].

**Table 4:** Predicted probabilities of good performance at different scores on the delivery-only index using Tanzania Round 1 (incl. Zanzibar) data

Delivery-only index score (% frequency) *n* = 282	Predicted probability (CI) of good performance – relative^a^	Predicted probability (CI) of good performance – absolute^b^
0 (0.71%)	< 0.001	< 0.001
1 (0.00%)	< 0.001	< 0.001 [< 0.001–0.001]
2 (0.71%)	< 0.001 [< 0.001–0.001]	< 0.001 [< 0.001–0.001]
3 (1.42%)	< 0.001 [< 0.001–0.002]	< 0.001 [< 0.001–0.002]
4 (6.38%)	0.001 [< 0.001–0.006]	< 0.001 [< 0.001–0.004]
5 (10.99%)	0.004 [0.001–0.014]	< 0.001 [< 0.001–0.006]
6 (12.06%)	0.012 [0.004–0.035]	0.001 [< 0.001–0.011]
7 (19.15%)^c^	0.041 [0.020–0.084]	0.002 [< 0.001–0.018]
8 (14.54%)	0.127 [0.081–0.195]	0.006 [0.001–0.032]
9 (17.02%)	0.332 [0.251–0.423]	0.016 [0.005–0.056]
10 (8.51%)	0.628 [0.501–0.740]	0.043 [0.017–0.104]
11 (3.55%)	0.852 [0.724–0.926]	0.110 [0.053–0.215]]
12 (3.19%)	0.951 [0.869–0.983]	0.252 [0.118–0.459]
13 (1.77%)	0.985 [0.943–0.996]	0.479 [0.209–0.761]

^a^Relative good performance is defined as being in the top 25% of the total QoC score distribution

^b2^Absolute good performance is defined as performing ≥80% of all observed routine L&D actions correctly; 2.84% of deliveries observed demonstrated absolute good performance

^c^Mean = 7.48, median = 7

## Discussion

This study compared a previously validated comprehensive index measuring intrapartum and immediate postpartum care process quality with a shorter index of items that can be assessed at or immediately after delivery, including ENC. Content and criterion validation of the 13-item index composed of “delivery-only” items supported its utility as a quality assessment measure. The comprehensive QoPIIPC index developed earlier represents more dimensions of QoPIIPC and appears to be a superior tool for classifying deliveries as poorly or well-performed. However, the delivery-only index represents a more parsimonious list of items and avoids several that are performed nearly universally. The delivery-only index is a robust and more feasible option for quality assessment in settings where complete episodes of L&D care cannot be observed due to resource constraints.

### Limitations and strengths

This study faced limitations related to the QoC Assessment data analyzed, such as a potential Hawthorne effect, lack of generalizability to facilities with a lower-volume of deliveries, and restriction of data to routine L&D/ENC interventions. These limitations have been reported in depth elsewhere [39]. However, considering the resources and effort required to observe L&D care even with the most efficient tools, it may be appropriate for the use of the delivery-only index to be restricted to higher-volume facilities. Additionally, the QoC Assessments were conducted across a diverse sample of facilities, from rural health centers to referral hospitals, possibly reducing the effect of non-random sampling on generalizability.

This study has a number of important strengths. Much research on obstetric QoC and its potential measurement has relied on routine data sources that are not designed for or suitable for quality assessment. Observations, such as those conducted in the QoC Assessments, may provide improvements in completeness, accuracy, and specificity [29–31]. This study is also one of very few to include validation of quality measures with empirical data from low income country settings, and the only one to focus on the time of delivery.

### Program and research implications

The delivery-only index may reduce the burden of observation sufficiently to enable periodic L&D care quality assessment at the facility level, complementing other clinical supervision and records-based monitoring activities. All users must be oriented to the fact that this tool is not intended to be a comprehensive clinical guideline, checklist, or job aid; however, it can be used to provide valid information on care quality through targeted observation and may address gaps that have been identified through global MNC research and monitoring.

As greater attention is paid to the fact that QoC must improve if the global targets for maternal and neonatal mortality reduction are to be achieved, understanding of the construct is evolving [48]. A recent study by Souza et al. concludes that coverage with life-saving interventions may be insufficient to reduce maternal deaths without improvements in overall care quality [49]. This nuanced understanding of QoPIIPC suggests that observation of care may be crucial in quality assurance and improvement (QA/QI). Key aspects of QoPIIPC, such as provider-patient interactions and provider vigilance of danger signs, are not captured in medical records and registers. Tools that bring observation out of the research setting and into programs are necessary to address gaps in knowledge about routine L&D care quality, particularly as most assessment of QoPIIPC has focused on adverse events such as deaths and near misses [50–52].

The need for valid quality assessment becomes particularly urgent as incentives to women for facility delivery, removal of user fees, performance-based financing for providers and health facilities, and other trends increase the use of facility-based L&D care [18–20, 53]. In-depth verification of QoC contributes to QA/QI initiatives and is essential when providers and facilities are paid for performance [54, 55]. Anecdotal and program evidence suggests that when specific actions are emphasized in policy, their performance may be affected in ways that cannot be detected through record review. For example, partographs may be filled in after labor if completed partographs are rewarded in performance-based financing programs [56]. An improved ability to efficiently measure QoPIIPC may also strengthen the validity of future research on quality assurance and improvement within maternal and newborn health services.

## Conclusions

The quality measure evaluated in this study provides a new tool that can be used to evaluate routine L&D care in health facilities more easily using clinical observation. There is increased global attention to the care provided to mothers and newborns at the time of delivery, the focus of this index. This index complements and addresses gaps in existing tools and may improve knowledge regarding the quality of MNC in sub-Saharan Africa and other low income country settings. Expanded quality assessment using validated tools may help programs target QI activities and promote further reductions in maternal and neonatal mortality and morbidity.

## Abbreviations

AUROC: Area under the receiver operating characteristic curve
EmONC: Emergency obstetric and newborn care
ENC: Essential newborn care
L&D: Labor and delivery
MCHIP: Maternal and Child Health Integrated Program
MNC: Maternal and newborn care
QA/QI: Quality assurance/quality improvement
QoC: Quality of care
QoPIIPC: Quality of the process of intrapartum and immediate postpartum care
UN: United Nations
USAID: United States Agency for International Development

## References

[1] Lozano R, Wang H, Foreman KJ, Rajaratnam JK, Naghavi M, Marcus JR (2011). Progress towards millennium development goals 4 and 5 on maternal and child mortality: an updated systematic analysis. Lancet.

[2] Hogan MC, Foreman KJ, Naghavi M, Ahn SY, Wang M, Makela SM (2010). Maternal mortality for 181 countries, 1980-2008: a systematic analysis of progress towards millennium development goal 5. Lancet.

[3] World Health Organization (2015). Trends in maternal mortality: 1990 to 2015. Estimates developed by WHO, UNICEF, UNFPA and the World Bank.

[4] Countdown to 2015 (2015). A Decade of Tracking Progress for Maternal, Newborn and Child: The 2015 Report.

[5] Lawn JE, Lee AC, Kinney M, Sibley L, Carlo WA, Paul VK (2009). Two million intrapartum-related stillbirths and neonatal deaths: where, why, and what can be done?. Int J Gynaecol Obstet.

[6] Hodgins S (2013). Achieving better maternal and newborn outcomes: coherent strategy and pragmatic, tailored implementation. Glob Health Sci Pract.

[7] World Health Organization (2006). Pregnancy, childbirth, postpartum and newborn Care: a guide for essential practice, integrated Management of Pregnancy and Childbirth Toolkit.

[8] Shankar A, Bartlett L, Fauveau V, Islam M, Terreri N (2008). Countdown to 2015 maternal health group. Delivery of MDG 5 by active management with data. Lancet.

[9] Campbell OM, Graham WJ (2006). Lancet maternal survival series steering group: strategies for reducing maternal mortality: getting on with what works. Lancet.

[10] Lawn JE, Kerber K, Enweronu-Laryea C, Massee Bateman O (2009). Newborn survival in low resource settings--are we delivering?. BJOG.

[11] Ronsmans C, Campbell O (2011). Quantifying the fall in mortality associated with interventions related to hypertensive diseases of pregnancy. BMC Public Health.

[12] Wall SN, Lee AC, Carlo W, Goldenberg R, Niermeyer S, Darmstadt GL (2010). Reducing intrapartum-related neonatal deaths in low- and middle-income countries-what works?. Semin Perinatol.

[13] Firoz T, Sanghvi H, Merialdi M, von Dadelszen P (2011). Pre-eclampsia in low and middle income countries. Best Pract Res Clin Obstet Gynaecol.

[14] Harvey SA, Blandón YC, McCaw-Binns A, Sandino I, Urbina L, Rodríguez C (2007). Nicaraguan maternal and neonatal health quality improvement group: are skilled birth attendants really skilled? A measurement method, some disturbing results and a potential way forward. Bull World Health Organ.

[15] Stanton C, Armbruster D, Knight R, Ariawan I, Gbangbade S, Getachew A (2009). Use of active management of the third stage of labour in seven developing countries. Bull World Health Organ.

[16] Wall SN, Lee AC, Niermeyer S, English M, Keenan WJ, Carlo W (2009). Neonatal resuscitation in low-resource settings: what, who, and how to overcome challenges to scale up?. Int J Gynaecol Obstet.

[17] Randive B, Diwan V, De Costa A (2013). India's conditional cash transfer Programme (the JSY) to promote institutional birth: is there an association between institutional birth proportion and maternal mortality?. PLoS One.

[18] Baral G (2012). An assessment of the safe delivery incentive program at a tertiary level hospital in Nepal. J Nepal Health Res Counc.

[19] Powell-Jackson T, Neupane BD, Tiwari S, Tumbahangphe K, Manandhar D, Costello AM (2009). The impact of Nepal's national incentive programme to promote safe delivery in the district of Makwanpur. Adv Health Econ Health Serv Res.

[20] World Health Organization (2003). Managing complications in pregnancy and childbirth: a guide for midwives and doctors.

[21] Maine D, Barzelatto J, Berer M, Sundari RTK (1999). What’s so special about maternal mortality?. Safe motherhood initiatives: critical issues.

[22] Donabedian A (1980). The definition of quality and approaches to its management, vol 1: Explorations in Quality Assessment and Monitoring.

[23] Donabedian A (1988). The quality of care. How can it be assessed?. JAMA..

[24] Tripathi Vandana (2015). A literature review of quantitative indicators to measure the quality of labor and delivery care. International Journal of Gynecology & Obstetrics.

[25] Hulton L, Matthews Z, Stones RW (2000). A framework for the evaluation of quality of care in maternity services.

[26] Bazant E, Rakotovao JP, Rasolofomanana JR, Tripathi V, Gomez P, Favero R (2013). Quality of care to prevent and treat postpartum hemorrhage and pre-eclampsia/eclampsia: an observational assessment in Madagascar's hospitals. Med Sante Trop.

[27] Bailey P, Paxton A, Lobis S, Fry D (2006). The availability of life-saving obstetric services in developing countries: an in-depth look at the signal functions for emergency obstetric care. Int J Gynaecol Obstet.

[28] Paxton A, Bailey P, Lobis S (2006). The United Nations process indicators for emergency obstetric care: reflections based on a decade of experience. Int J Gynaecol Obstet.

[29] Hermida J, Nicholas DD, Blumenfeld SN (1999). Comparative validity of three methods for assessment of the quality of primary health care. Int J Qual Health Care.

[30] Leonard KL, Masatu MC (2005). The use of direct clinician observation and vignettes for health services quality evaluation in developing countries. Soc Sci Med.

[31] Broughton EI, Ikram AN, Sahak I (2013). How accurate are medical record data in Afghanistan's maternal health facilities? An observational validity study. BMJ Open.

[32] Duffy S, Crangle M (2009). Delivery room logbook - fact or fiction?. Trop Dr.

[33] Hofer TP, Bernstein SJ, DeMonner S, Hayward RA (2000). Discussion between reviewers does not improve reliability of peer review of hospital quality. Med Care.

[34] Localio AR, Weaver SL, Landis JR, Lawthers AG, Brenhan TA, Hebert L (1996). Identifying adverse events caused by medical care: degree of physician agreement in a retrospective chart review. Ann Intern Med.

[35] Smith MA, Atherly AJ, Kane RL, Pacala JT (1997). Peer review of the quality of care. Reliability and sources of variability for outcome and process assessments. JAMA..

[36] World Health Organization (2006). Managing obstructed and prolonged labor: education materials for teachers of midwifery.

[37] Goga AE, Muhe LM (2011). Global challenges with scale-up of the integrated management of childhood illness strategy: results of a multi-country survey. BMC Public Health.

[38] Manzi F, Schellenberg JA, Hutton G, Wyss K, Mbuya C, Shirima K (2012). Human resources for health care delivery in Tanzania: a multifaceted problem. Hum Resour Health.

[39] Tripathi V, Stanton C, Strobino D, Bartlett L (2015). Development and validation of an index to measure the quality of facility-based labor and delivery care processes in sub-Saharan Africa. PLoS One.

[40] UNICEF. The State of the World's Children 2016 statistical tables. https://data.unicef.org/resources/state-worlds-children-2016-statistical-tables/. Accessed 10 Apr 2019.

[41] UNICEF. Levels and Trends in Child Mortality. Report 2010. Estimates developed by the UN inter-Agency Group for Child Mortality Estimation. New York: UNICEF. p. 2010.

[42] World Health Organization (2011). Atlas of Health Statistics of the African Region, 2011.

[43] ACCESS Project (2008). Best practices in maternal and newborn Care: a learning resource package for essential and basic emergency obstetric and newborn Care.

[44] Prevention of Postpartum Hemorrhage Initiative. AMTSL survey: tools to conduct a national survey. https://www.k4health.org/toolkits/postpartumhemorrhage/amtsl-survey-tools-conduct-national-survey. Accessed 10 Apr 2019.

[45] MCHIP. Maternal and Newborn Quality of Care Surveys. https://www.mchip.net/qocsurveys/. Accessed 10 Apr 2019.

[46] Akobeng AK (2007). Understanding diagnostic tests 3: receiver operating characteristic curves. Acta Paediatr.

[47] Hanley JA, McNeil BJ (1982). The meaning and use of the area under a receiver operating characteristic (ROC) curve. Radiology..

[48] Tunçalp Ӧ, Were WM, MacLennan C, Oladapo OT, Gülmezoglu AM, Bahl R, Daelmans B, Mathai M, Say L, Kristensen F, Temmerman M, Bustreo F (2015). Quality of care for pregnant women and newborns-the WHO vision. BJOG..

[49] Souza JP, Gülmezoglu AM, Vogel J, Carroli G, Lumbiganon P, Qureshi Z (2013). Moving beyond essential interventions for reduction of maternal mortality (the WHO multicountry survey on maternal and newborn health): a cross-sectional study. Lancet..

[50] Drife JO (2006). Perinatal audit in low- and high-income countries. Semin Fetal Neonatal Med.

[51] Kongnyuy EJ, Uthman OA (2009). Use of criterion-based clinical audit to improve the quality of obstetric care: a systematic review. Acta Obstet Gynecol Scand.

[52] Pirkle CM, Dumont A, Zunzunegui MV (2011). Criterion-based clinical audit to assess quality of obstetrical care in low- and middle-income countries: a systematic review. Int J Qual Health Care.

[53] Lim SS, Dandona L, Hoisington JA, James SL, Hogan MC, Gakidou E (2010). India's Janani Suraksha Yojana, a conditional cash transfer programme to increase births in health facilities: an impact evaluation. Lancet..

[54] Rusa L, Ngirabega Jde D, Janssen W, Van Bastelaere S, Porignon D, Vandenbulcke W (2009). Performance-based financing for better quality of services in Rwandan health centres: 3-year experience. Tropical Med Int Health.

[55] Eichler R, Auxila P, Pollock J. Flagship Program on Health Sector Reform and Sustainable Financing: Performance-based payment to improve the impact of health services: Evidence from Haiti. Washington, DC: World Bank; 2001. https://www.eldis.org/document/A29779. Accessed 10 Apr 2019.

[56] Fistula Care and Maternal Health Task Force (2012). Revitalizing the partograph: does the evidence support a global call to action?—report of an expert meeting, New York, November 15–16, 2011.

